# Rapid, Self-driven Liquid Mixing on Open-Surface Microfluidic Platforms

**DOI:** 10.1038/s41598-017-01725-0

**Published:** 2017-05-11

**Authors:** Jared M. Morrissette, Pallab Sinha Mahapatra, Aritra Ghosh, Ranjan Ganguly, Constantine M. Megaridis

**Affiliations:** 1 Department of Mechanical and Industrial Engineering, University of Illinois at Chicago, Chicago, IL 60607-7022 United States; 2 Department of Power Engineering, Jadavpur University, Kolkata, 700098 India

## Abstract

Self-driven surface micromixers (SDSM) relying on patterned-wettability technology provide an elegant solution for low-cost, point-of-care (POC) devices and lab-on-a-chip (LOC) applications. We present a SDSM fabricated by strategically patterning three wettable wedge-shaped tracks onto a non-wettable, flat surface. This SDSM operates by harnessing the wettability contrast and the geometry of the patterns to promote mixing of small liquid volumes (µL droplets) through a combination of coalescence and Laplace pressure-driven flow. Liquid droplets dispensed on two juxtaposed branches are transported to a coalescence station, where they merge after the accumulated volumes exceed a threshold. Further mixing occurs during capillary-driven, advective transport of the combined liquid over the third wettable track. Planar, non-wettable “islands” of different shapes are also laid on this third track to alter the flow in such a way that mixing is augmented. Several SDSM designs, each with a unique combination of island shapes and positions, are tested, providing a greater understanding of the different mixing regimes on these surfaces. The study offers design insights for developing low-cost surface microfluidic mixing devices on open substrates.

## Introduction

Microfluidic devices capable of achieving complex liquid-handling tasks, such as transport, metering, separation and mixing, have found niche applications in numerous fields, including point-of-care (POC) diagnostics^1^, lab-on-a-chip (LOC) applications^2–5^, and micro total analysis systems (μTAS)^6^. Although most conventional microfluidics devices have historically deployed flow-through systems, a more recent strategy of handling liquid samples in the form of *discrete* droplets has gained popularity^7^. Droplet-based microfluidics is advantageous over flow-through microfluidics, since the liquid sample handling in the former is relatively free from the common problems of the latter, such as axial dispersion, sample dilution, and cross-contamination^8, 9^. In droplet-based microfluidics, individual (or sequences of) droplets of the sample liquid may be handled either within an immiscible liquid in a closed microchannel^10^ or on open surfaces^11^. The dispensed liquid volumes range from 1 μL to 1 mL; the lower range is common to many microfluidic applications^12^, while the larger volumes are relevant to on-chip liquid storage^13^, or some specialized microfluidic applications that require larger samples (*e*.*g*. whole-blood assays^14, 15^). Open-surface type microfluidic devices offer the possibility of low-cost fabrication –these devices can be built on low-cost, paper or plastic surfaces and do not require elaborate fabrication of embedded microchannels – and hence, are ideally suited for POC diagnostics^16^.

Like the flow-through microfluidic devices, rapid and efficient mixing is also an essential pre-requisite for open-surface microfluidic platforms. Open-surface micromixers may be of *active* or *passive* type, depending on whether external energy input is required or not, respectively. Active micromixer designs for surface microfluidics have recently been adopted by several groups in the community: for example, mixing using electrowetting on dielectric (EWOD)^17^, magnetic^18, 19^, dielectrophoretic^20^, surface acoustic wave (SAW)^21^, or thermocapillary actuation^11^ has been found to be effective in controlled manipulation of discrete liquid volumes on solid substrates. However, these devices are complex, since they also include off-chip components (*i*.*e*. function generators, voltage sources, electromagnets, *etc*.) that require external power input. This requirement adds to the net cost and operational complexity of the micromixer, thereby limiting the deployment of energy-consuming mixers as a platform for low-cost, POC diagnostics. Therefore, the need to develop a passive micromixer, which on one hand, shuns off-chip dependency and laborious fabrication techniques, and at the same time is effective, simple and inexpensive, cannot be overemphasized.

Harnessing the surface-tension forces for inducing liquid transport has been seen as an attractive option for open-surface microfluidic devices. Recent advancements in paper-based microfluidic devices^22^ have produced low-cost, simple solutions to most microfluidic tasks, such as liquid transport, sample separation (*e*.*g*. plasma from whole blood), and in some cases, diffusive mixing^16, 23^. Although these devices are inexpensive and offer *pumpless* transport, rapid, advective mixing of liquids is still a paramount challenge. Liquid transport on paper is typically slow, as it is driven via capillary action through a porous network. Traditional paper-based microfluidic devices work very well when a sample liquid imbibes through a permeable segment of the paper substrate (pre-impregnated with analyte chemicals) and heterogeneously reacts to produce a simple colorimetric detection signal^22^. However, these designs cannot perform higher-order liquid transport tasks, such as metered transport and advective homogeneous (liquid phase) mixing.

Surface tension-driven micromixers, whether on open^24–26^ or semi-open^27, 28^ configurations, have been proposed in the literature. These devices have attained liquid transport speeds up to 10 mm/s and mixing time scales of about 1 s. The simplest biochemical protocols on surface microfluidic platforms may work well with spot mixing, where the sample is deposited directly on a porous substrate that is pre-suffused with the appropriate analytes or diagnostic chemicals^29^; but this cannot be extended to more complex protocols that require multiple intermediate steps. Surface microfluidics-based rapid diagnostic strips mostly deploy a separate liquid uptake zone and a testing zone for convenience of sample dispensing and signal readout^30, 31^; sometimes zone-segregation is adopted to prevent premature sample degradation prior to detection^32^. In specific applications, the sample and the participating reagent in the biochemical protocol mix and react *in situ* before contacting a third reagent that is used for detection (*e*.*g*. colorimetric, electrochemical, fluorescent, *etc*.)^33, 34^. For such systems, a robust surface microfluidic mixer should have the capability of separated uptake (of sample and reagents), precise metering (the requisite volume needed for the specific protocol), merging (the sample and reagent), mixing and transporting (to the detection zone). Achieving all of these complex tasks on a planar surface *without* external power input has remained elusive so far.

Recent advancements in rapid, pumpless, liquid transport on wettability-patterned open-air surfaces^35–39^ have motivated the design of passive microfluidic devices that can mix liquids efficiently without the limitations associated with traditional paper-based and closed-channel micromixers. Here we present an innovative, passive, open micromixer design, *a self-driven surface micromixer* (*SDSM*), capable of rapid, pumpless transport (at speeds ~100 mm/s), and fast, homogenous mixing (within ~100 ms). Our SDSM harnesses surface-tension forces using strategically-patterned, highly wettable (superhydrophilic) wedge-shaped tracks on a non-wettable (superhydrophobic) substrate (*e.g.*, a hydrophobized paper or plastic film) to induce advective mixing of two aqueous liquids that are confined within the wettable domains. The wettability contrast between the superhydrophilic regions and the surrounding superhydrophobic background leads to capillary-driven, directional liquid transport along the superhydrophilic tracks. Sample liquids, dispensed at ~5 μL dropwise increments at the narrow ends of two juxtaposed superhydrophilic tracks, are first transported to a coalescence station at the wide ends of the wedge tracks. Upon exceeding a pre-determined volume, the dispensed liquid samples that grow adjacent to each other eventually coalesce, initiating the mixing stage. The merged volume contacts a third, superhydrophilic track that transports it further away downstream, inducing advective mixing. The mixed liquid collects at the “end reservoir” – a superhydrophilic patch that may serve as the detection zone in a biosensor application. Liquid mixing is also facilitated by introducing two-dimensional, superhydrophobic “islands” as obstacles within the superhydrophilic mixing zone of the third track. These non-wettable islands alter and even deflect the flow to enhance mixing, akin to what three-dimensional obstacles^40–43^ do in closed microchannels. High transport speeds enable rapid mixing in the SDSM, while a few specific configurations of the superhydrophobic islands enhance mixing homogeneity. Liquid mixing on various SDSM designs is investigated and the mixing efficiencies of these designs are compared through quantitative image analysis.

It is important to note that the main focus of the present paper is to demonstrate mixing on the SDSM at time scales more suitable for standard POC protocols. Microfluidic paper-based analytical systems reported in the literature involve biochemical reaction time ranging from 1 s to a few minutes^44, 45^. However, in most of these paper-based microfluidics assays, imbibition-transport through paper takes approximately 4–5 minutes^46^, which affects the response time and the detection speed of the device. Our SDSM cuts down the transport and mixing times significantly (~1 s); this not only improves the response time, but also reduces the problems associated with sample evaporation and dryout in paper-based assays. Further design modifications of each SDSM, based on specific POC protocols, would require investigating the actual biochemical reactions on the SDSM, and thus are left as a future exercise.

## Results and Discussion

### SDSM Design Configurations

**Figure 1:**
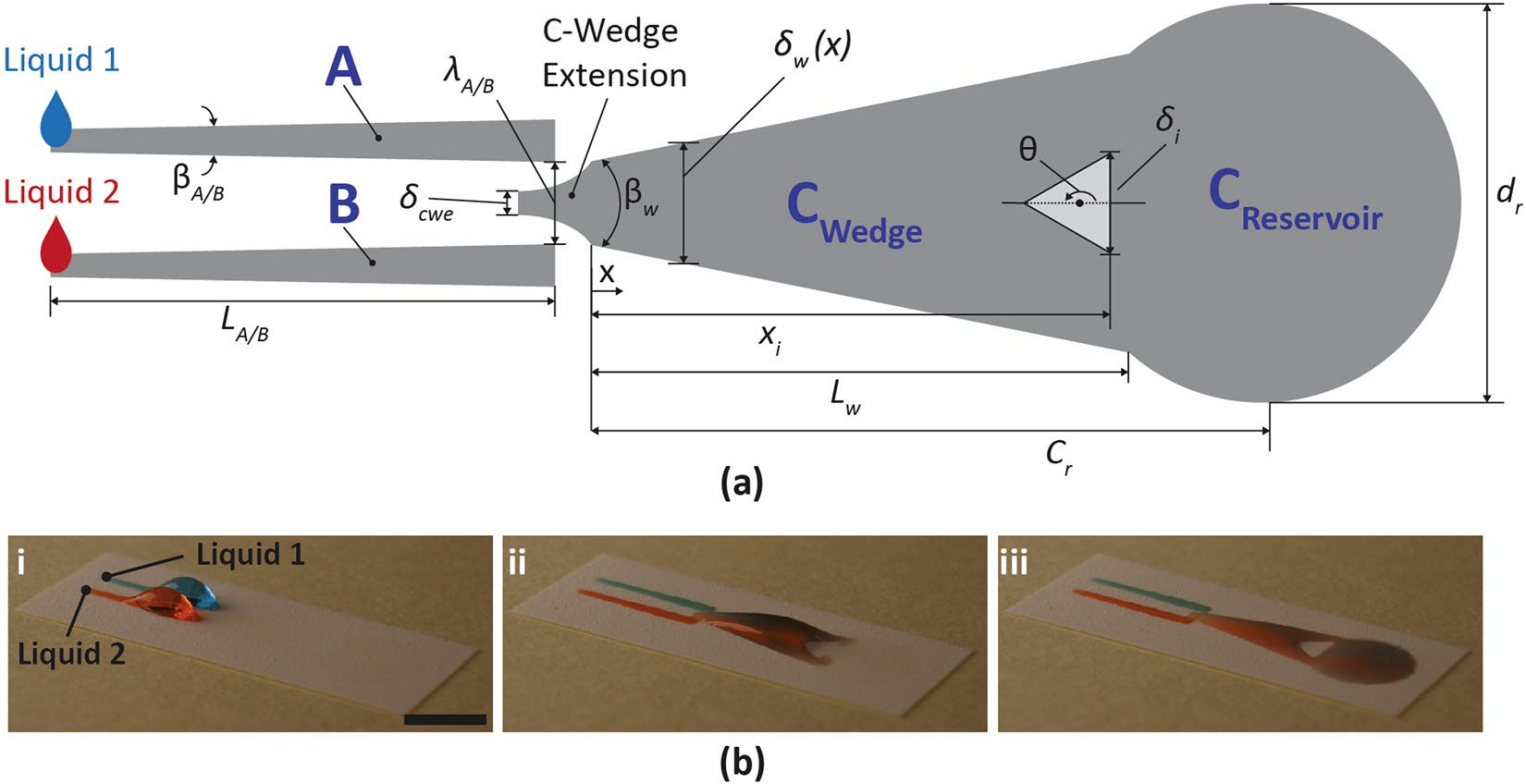
(**a**) Schematic depiction of the basic SDSM configuration ﻿studied herein. The superhydrophobic island within C_wedge_ can be square, circular or triangular with different orientation angles (θ = 0 to 180°). In general, *δ*_*i*_ was taken at the widest part of the island (for details see Fig. S1 of the SI). (**b**) Representative images of a tested SDSM: (i) before, (ii) during, and (iii) after mixing of two liquids. To show the concept, dyed water droplets (Liquid 1 and Liquid 2) were dispensed in tracks A and B. The scale bar in (i) denotes 1 cm, and applies to all images in (**b**). For more details on the mixing process, see video SM1 of the SI.

A top-view schematic of the SDSM is shown in Fig. 1a. The mixer features two juxtaposed, identical, wedge-shaped, superhydrophilic tracks (A and B), along with a third wedge-shaped superhydrophilic track (C). Tracks A and B are ~12.25 mm long, 1.05 mm at their narrower ends, and have wedge angles β_A_ = β_B_ = 2.2°. The wider ends of these tracks have an edge-to-edge distance *λ* = 2 mm. The superhydrophilic C-track has three regions; the C-wedge extension (left), the transport-track (*C*_*Wedge*_), and the circular reservoir (*C*_*Reservoir*_). The C-wedge extension at the beginning of the C-track has an edge width *δ*_*cwe*_ = 1 mm and protrudes into the superhydrophobic region between the A and B tracks by approximately 1 mm; it is also centered between the two tracks (as shown in Fig. 1a). The *C*_*Wedge*_ has a width of 2 mm at *x* = 0, an apex angle β_w_ ≈ 22.8° and is approximately 12.2 mm in length (*L*_*w*_). The center of the reservoir sits at *C*_*r*_ ~ 15.2 mm from x = 0, and has a diameter *d*_*r*_ ~ 9.1 mm. These dimensions were designated after extensive experimentation on the size of the different components of the mixer.

With the exception of the control case, the C-track of each SDSM design was patterned with a superhydrophobic “island.” The SDSM used in this work featured similar track designs (*i*.*e*. tracks A, B, and C); they only differed with regards to the design of the superhydrophobic islands patterned on the *C*_*Wedge*_. The relevant design parameters, as marked in Fig. 1a, include the island shape, orientation (*θ*), area ratio (*α*, Eq. 1), and constriction ratio (δ*, Eq. 2). The ratio of the surface area of the hydrophobic island (*A*_*i*_) to the overall surface area (*A*_*w*_ = 114 mm^2^ for all designs) of the hydrophilic *C*_*Wedge*_ is

1 $$\alpha =\frac{{A}_{i}}{{A}_{w}}.$$

The constriction ratio (δ*) is the fraction of the width of the widest part of the island *δ*_*i*_ and the width *δ*_*w*_ of track C at the axial position (*x*_*i*_) where the island is widest (see Fig. 1a).

2 $${\delta }^{\ast }=\frac{{\delta }_{i}}{{\delta }_{w}(x={x}_{i})}$$

Various SDSM configurations having a superhydrophobic island on the superhydrophilic *C*_*Wedg*e_ (22 in total) were studied and are listed in Table 1. An SDSM without a superhydrophobic island was considered as the control case, and mixing on all other SDSM described in Table 1 are compared with it.

**Table 1:** Various SDSM configurations and associated design parameters, as constructed using design of experiments (DOE) criteria (see Methods Section below).

Configuration	Island Shape	θ (°)	*α*	δ*	Configuration	Island Shape	θ (°)	*α*	δ*
1	Triangle	90	0.02	0.4	12	Square	0	0.04	0.5
2	Circle	0	0.02	0.5	13	Circle	0	0.04	0.6
3	Triangle	0	0.02	0.6	14	Square	0	0.04	0.6
4	Square	0	0.02	0.6	15	Circle	0	0.05	0.4
5	Square	0	0.03	0.4	16	Triangle	180	0.05	0.4
6	Circle	0	0.03	0.4	17	Square	0	0.05	0.5
7	Triangle	90	0.03	0.5	18	Circle	0	0.05	0.6
8	Square	45	0.03	0.6	19	Square	45	0.06	0.4
9	Triangle	45	0.03	0.6	20	Circle	0	0.06	0.4
10	Triangle	0	0.04	0.4	21	Circle	0	0.06	0.5
11	Triangle	45	0.04	0.5	22	Triangle	180	0.06	0.6

The control case (SDSM without island) is not listed in this table.

### Liquid Transport and Mixing on the SDSM

Liquid transport is initiated entirely by capillary force; the ensuing flow induces advective-diffusive mixing on the SDSM, as demonstrated in Fig. 1b, where dyed water (blue, red) was used to demonstrate the working principle. The fluid transport is initiated by dispensing droplets of two probe liquids (*e*.*g*. Liquid 1 and Liquid 2) onto the narrow ends of the feeding tracks A and B. As reported in our earlier work^36^, the tapered nature of the wettability-confined, wedge-shaped tracks generates a Laplace pressure gradient, which subsequently transports the liquid droplets from the narrow end to the wider end of each track (left to right in Fig. 1b). The Laplace pressure gradient developed in the liquid (surface tension *σ*) on a wedge-shaped track of apex angle β_*w*_, local width *δ*_*w*_ and an average apparent contact angle *θ*_*avg*_ at the wettability contrast line (which confines the liquid in transverse direction) scales as^36^

3 $$\frac{dP}{dx}\sim 2\sigma {\beta }_{w}/{\delta }_{w}^{2}\,\sin \,{\theta }_{avg}.$$

Upon reaching the wider ends of the tracks, liquid bulges are formed (Fig. 1b-i), which grow upon further droplet addition onto each track. As liquid accumulates at the wider end of each respective track (A or B), the liquid bulges eventually grow large enough laterally to touch and coalesce (~30 µL ammonium thiocyanate and ~40 µL ferric chloride), thus forming a liquid bridge^36^ over the intervening region between tracks A and B (where the C-wedge extension is laid). The droplet coalescence leads to a transversely inward and axially elongating flow within the liquid bridge – such flow has been found to be dominated by capillary and inertial forces^36, 47^. As the two liquid bulges oscillate after merging, they touch the underlying C-wedge extension and get transported down the superhydrophilic track (Fig. 1b-ii) by capillary pressure gradient (in a manner similar to the liquid transport on tracks A and B). The mixing liquids ultimately reach the *C*_*Reservoir*_ area (Fig. 1b-iii). Starting from coalescence, the process of mixing persists throughout these phases. By the time the liquid reaches *C*_*Reservoir*_, the same detaches (de-bridges) from the narrow ends of tracks A and B, leaving behind small residual volumes on each track.

The above design harnesses surface tension forces and converts them to rapid fluid movement, which translates into more efficient mixing of the two liquids. The Laplace pressure within the droplets (radius *r*) at the end of each track scales as *σ/r* ^48^. The kinematic pressure (denoting the inertial force) of the liquid on track C scales as *ρU* ^2^, where *ρ* denotes the liquid density. A balance between the capillary and inertial forces indicates that the induced velocity on track C would be *U* ~ (*σ/ρr*)^1/2^ or about 0.1 m/s for the conditions investigated herein. This is also corroborated by the experimentally observed velocity (~0.2 m/s) of the propagating liquid front on track C (see Fig. S2 of the SI). Assuming an average thickness *h* ~ 1 mm for the liquid film on track C, the pertinent flow Re ~ O(100), confirming the dominance of advection in the present SDSM configuration. Consequently, the mixing efficiency of the basic design (no island) is expected to be far better than a diffusion-driven mixing device. The presence of the islands is explored in the following to determine by how much these islands can further improve or accelerate the mixing processes, which may be important when high performance is required.

**Figure 2:**
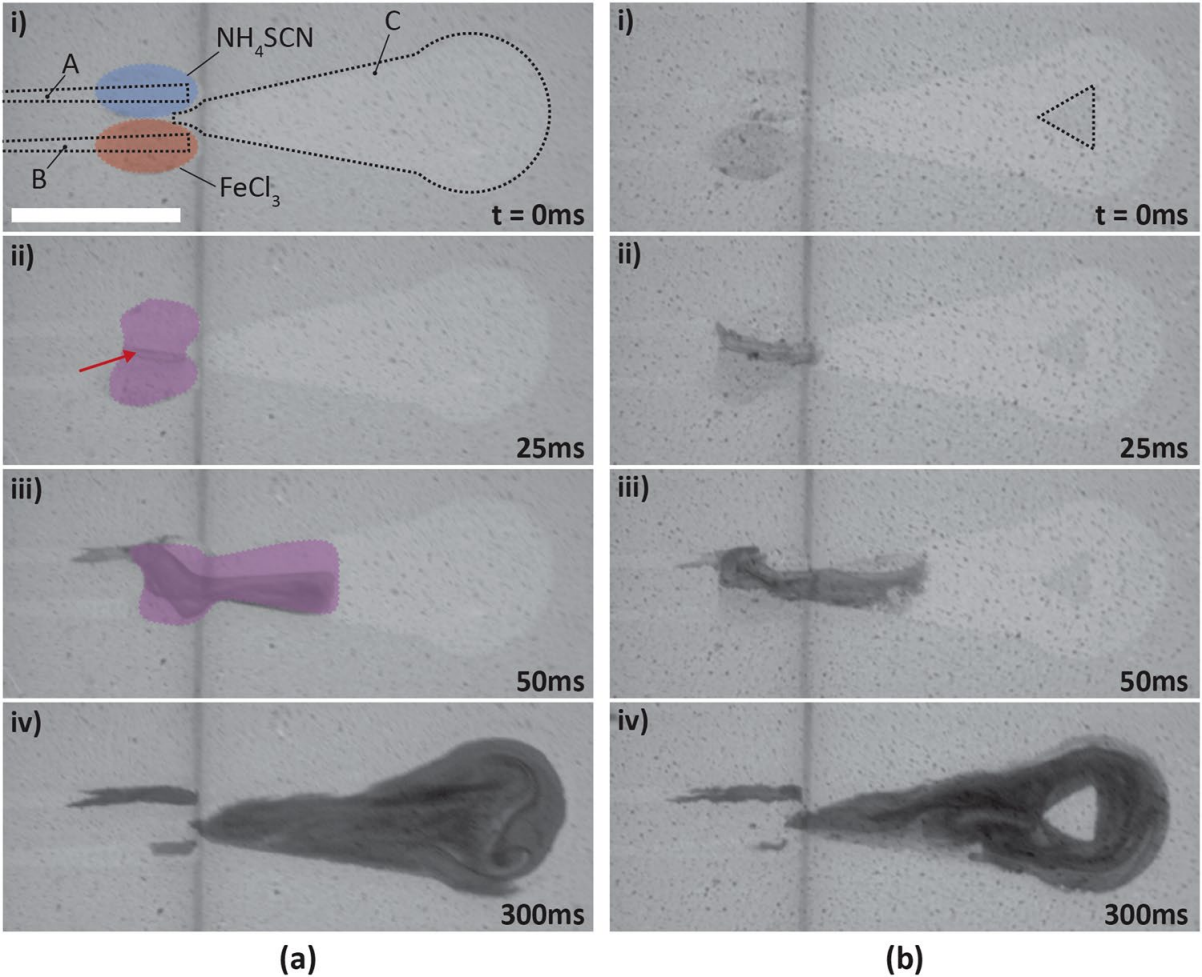
Mixing on two SDSM. (**a**,**b**) Timestamps displaying mixing of ~30 µL of ammonium thiocyanate (Liquid 1 on track A) and ~40 µL of ferric chloride (Liquid 2 on track 2) solutions on (**a**) a SDSM without a superhydrophobic island (control case), and (**b**) an identical SDSM with a triangular superhydrophobic island (outlined with a dotted-line in (**b**-**i**)).(i) Liquid bulges are formed as the two liquids accumulate at the wide ends of the A and B tracks; (ii) a liquid bridge is formed over the C wedge extension (mixing layer shown by red arrow); (iii) the solutions mix and are driven pumplessly to the right into the C_Wedge_ area; (iv) further mixing is accomplished with advective transport along C_wedge_ and around the superhydrophobic island (if present). The scale bar in the lower left corner of (**a**-**i**) denotes 1 cm and applies to all images in (**a**) and (**b**). The transparent red and blue ovals in (**a**-**i**), and the transparent purple shapes in (**a**-**ii**, **a**-**iii**) have been overlaid onto the images for demonstration purposes, to demarcate the boundary of the liquid bulge. In addition, the grey vertical streaks in all frames are markings on the underlying supporting platform used to align the SDSM during imaging, and had no influence on the mixing process.

Detailed images of the droplet-coalescence sequence, liquid bridge formation, and liquid transport onto C-wedge is shown in Fig. 2. Here, aqueous solutions of ammonium thiocyanate (NH_4_SCN, Sigma Aldrich, reagent grade 97.5%; 0.5 M in H_2_O) and ferric chloride (FeCl_3_, Sigma Aldrich, reagent grade 97%; 0.25 M in H_2_O) were used. Since these solutions are aqueous, their wetting behavior on the SDSM is similar to that of pure water, *i*.*e*. the contact angles for the NH_4_SCN and FeCl_3_ solutions on the superhydrophobic region of the SDSM are ${\theta }_{N{H}_{4}SCN}^{\ast } \sim 159^\circ \pm 2.5^\circ$ and ${\theta }_{FeC{l}_{3}}^{\ast } \sim 160^\circ \pm 1^\circ$ (see Figure S3 of the SI), and both solutions have ~0° contact angle in the UV-exposed, superhydrophilic region. These two solutions were used to aid in mixing characterization, since upon homogeneous mixing, they produce a sharp color change^49^ — from transparent to reddish brown (due to the generation of ferric thiocyanate [Fe(SCN)_6_]^3−^). This drastic color change was used to evaluate mixing with image analysis, and ultimately quantify the mixing homogeneity of the SDSM designs. The top-view images of each mixing event were captured every millisecond using a high-speed camera (Phantom M310; Vision Research). Generally, there are challenges associated with quantifying mixing using image analysis, such as the need for high-contrast, high-quality imaging and lighting^50^, however, these are overcome by using transmitted light (LitePad HO+, 76 mm diameter, Daylight-5800°K; Rosco Labs); the dark well-mixed and the transparent unmixed solutions provided adequate contrast on the translucent SDSM. Transmitted-light measurements are preferred for mixing calculations, as they are unbiased when it comes to correctly quantifying mixing homogeneity in cases such as stratified mixing, *e*.*g*. when a thin, well-mixed (dark) layer overlays an unmixed (light) layer.

As mentioned earlier, the mixing process begins with coalescence of the liquid volumes which accumulate at the adjacent wide ends of tracks A and B. The two-component liquid thus comes in contact with the *C*_*Wedge*_ tip and is subsequently transported along the *C*_*Wedge*_ domain (see Video SM1 in the SI). Advective mixing continues as the two liquids advance through *C*_*Wedge*_ (Fig. 2a-iii) due to hemi-wicking and the Laplace pressure gradient. While being transported along *C*_*Wedge*_, the two liquids mix, forming dark brown laminae (mixing layers resulting from the reacted liquids). With time, the laminae eventually disperse, but still display areas of incomplete mixing (Fig. 2a-iv). For SDSM with a superhydrophobic island, liquid debridging, transport and mixing upstream of the islands occur just as in the control case (see Fig. 2b-ii,iii). However, the interaction between the liquids and the superhydrophobic island brings about flow attributes around and downstream of the island that were found to promote mixing (Fig. 2b-iv). The final mixing stage on a SDSM occurs when the liquid front reaches the far end of the wettability-confined *C*_*Wedge*_ and spreads over the *C*_*Reservoir*_ region, generating additional transverse vortices. As the liquid separates from tracks A and B into the narrow end of *C*_*Wedge*_, a small amount of residual volume is observed over the tracks A and B. Gravimetric measurements, conducted by absorbing these residual liquid volumes in small chips of pre-weighed super-absorbent porous substrates, indicate an average cumulative (tracks A and B) residual volume of ~3.1 + 0.03% of the liquid delivered to track C after each cycle of operation. This amount is quite low compared to the overall delivered volume, and thus has no ramifications for the reliability of the mixer.

### Influence of Island Parameters on Mixing Efficiency

**Figure 3:**
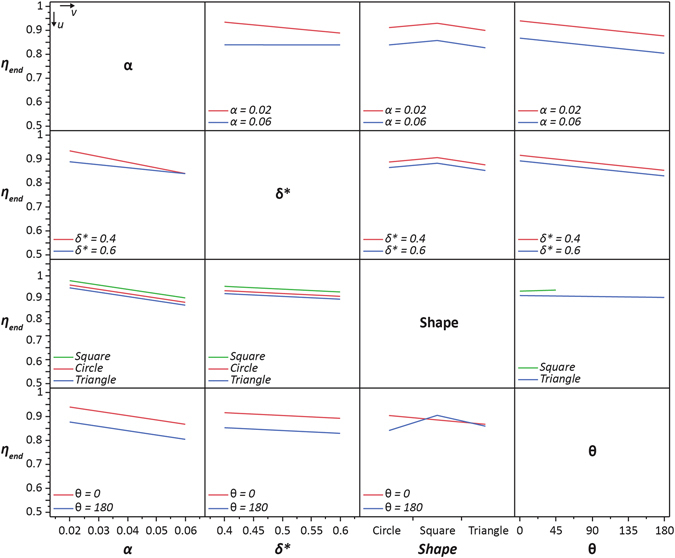
Interaction plot obtained by DOE data analysis of all SDSM configurations listed in Table 1. Here, JMP® statistical software was used to show interactions between the four island parameters (*α*, δ*, shape, or *θ*) and their effects on the mixing efficiency (*η*_*end*_) at the end of C_Wedge_ at t = 1 s. The plot consists of 12 sub-plots (indexed by *u* and *v*) that show how the mixing efficiency varies with the *v* ^th^ island parameter for fixed values of the *u* ^th^ island parameter. For example, the sub-plot (1,2; *i.e*. *u* = 1,*v* = 2) shows how *η*_*end*_ varies as a function of the constriction ratio (δ*) for two area ratios of *α* = 0.02 – red, or 0.06 – blue. Similarly, the sub-plot (3,4) shows how *η*_*end*_ varies with island orientation (*θ*) for fixed island shapes (square – green, triangle – blue). A detailed explanation of how these plots were generated can be found in section S4 of the SI.

The mixed-fluid homogeneity was quantified by calculating the mixing efficiency (*η*) at various time instances and locations along the C-track. The mixing efficiency for all SDSM (including the control case) was calculated at *x*_*end*_, a cross section approximately 1mm from the farthest end of the *C*_*Reservoir*_. For SDSM that featured a superhydrophobic island, the mixing efficiency was also calculated at *x*_*u*_ and *x*_*d*_, locations approximately 500 μm upstream and downstream of the island, respectively. Figure 3 shows how each island parameter influenced the mixing efficiency through an interaction plot^51^ comprised of 12 sub-plots, each demonstrating how two given island parameters together influence the final (*t* = 1 s) mixing efficiency (*η*) at the end of the *C*_*Reservoir*_ (*η*_*end*_ @ *x*_*end*_). Each sub-plot is indexed (*u*,*v*), and shows how the mixing efficiency varies with the *v* ^*th*^ island parameter (along respective horizontal axis) for specific values of the *u* ^*th*^ island parameter (each shown by a different curve). Thus, *u* or *v* = 1, 2, 3, 4 correspond to *α*, δ*, island shape, or *θ*, respectively. For example, sub plot (1, 2) (*i*.*e*. row 1, column 2 in Fig. 3) shows the variation of *η*_*end*_ with δ* for two distinct values of *α* (*i*.*e*., 0.02 and 0.06). Similarly, in sub-plot (3, 4), the mixing efficiency for specific island shapes (*square*, *triangle*) is plotted with respect to island orientation (*θ*). Figure 3 provides useful qualitative information regarding how the mixing efficiency with one specific island parameter is more or less strongly influenced by the values of other island parameters. While the mixing efficiency variations in Fig. 3 are strongly influenced by certain island parameters (strong interaction), other island parameters show far smaller influence. For example, observing the trends in row 1, one notes that regardless of the values of δ*, island shape, or *θ*, the mixing efficiency is always higher for the smaller area ratio, *α* = 0.02. Similarly, in row 2, although not as pronounced as the area-ratio correlations, the mixing efficiency is always higher for the lower constriction ratio (δ* = 0.4).

In general, curves that are *non-parallel* in Fig. 3 indicate that the two respective island parameters within each associated subplot strongly influence one another^51^ – implying they have a coupled effect on mixing efficiency (for more details, see section S4 of the SI). In essence, this means that unique combinations of these island parameters can influence the mixing efficiency of a SDSM depending upon the combination chosen. This is most evident in the α-δ* subplots (*e*.*g*. *u* = 1, *v* = 2 or *u* = 2, *v* = 1), where low area ratios and low constriction ratios contribute to better mixing, but depending on the combination of these two parameters the mixing efficiency can change. For instance, for an island with area ratio *α* = 0.06, the mixing efficiency remains the same regardless of the constriction ratio. But *η*_*end*_ shows a declining trend with rising δ* when *α* = 0.02. For the subplots of Fig. 3 where the *η*_*end*_ curves are *parallel* – as seen in subplots (1, 3), (1, 4), (2, 3), (2, 4), (3, 1), (3, 2), (4, 1), (4, 2), and to a lesser extent subplots (3, 4) and (4, 3) – it is evident that the two associated island parameters do not influence each other. For example, for *u* = 1–2, *v* = 4 (*i*.*e*., *α-θ*, and δ*-θ plots), the parallelism implies that the variation of *η*_*end*_ with *θ* is relatively insensitive to combinations of *α* and δ*. Instead, these two plots suggest that low area ratios and low constriction ratios contribute to better mixing efficiency for all values of *θ*, although mixing is best when the orientation angle is low.

### Liquid Movement around a Superhydrophobic Island

**Figure 4:**
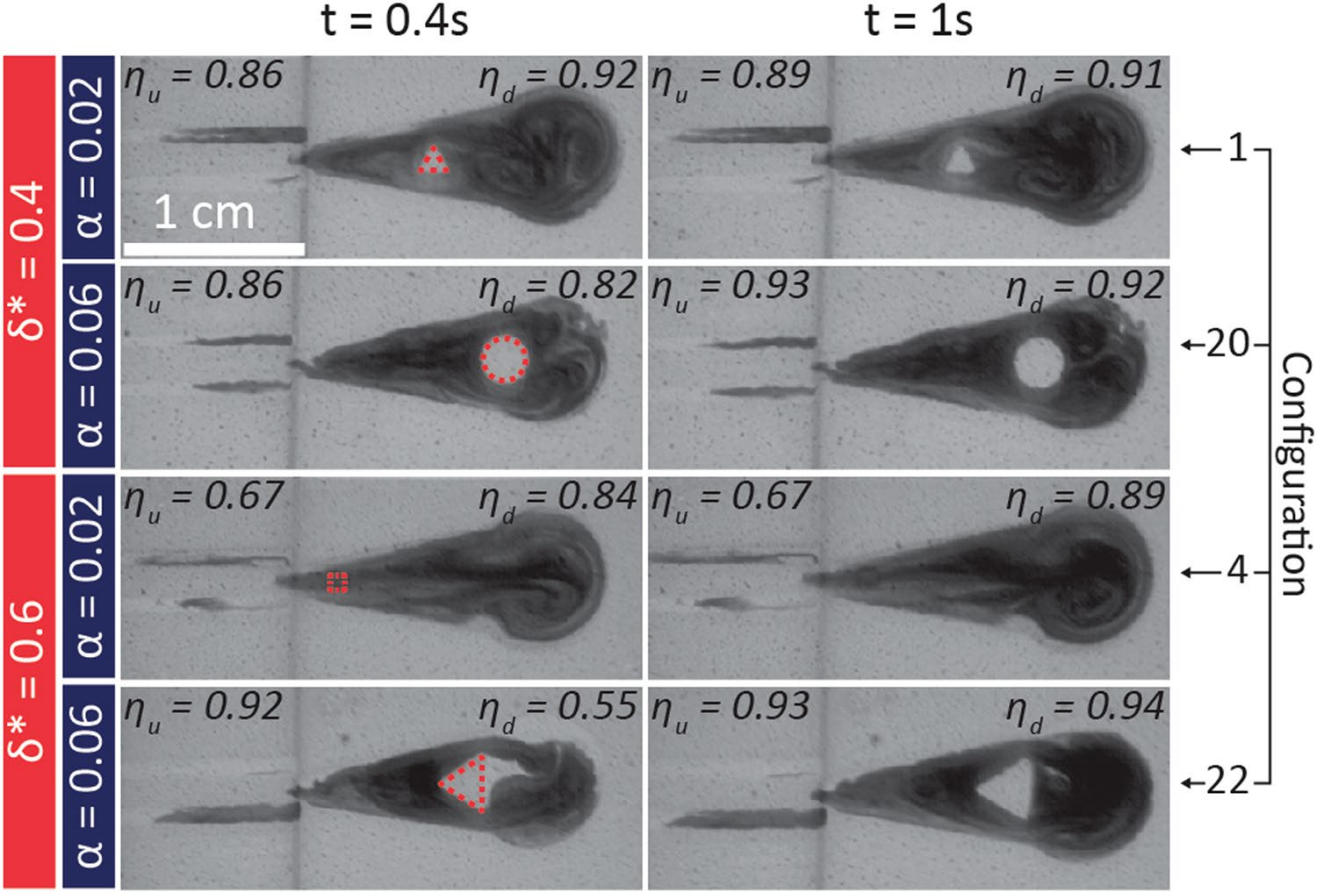
Images of transient mixing patterns near superhydrophobic islands for four select SDSM configurations. For t = 0.4 s (left column), high and low values of the area ratio (*α*) and constriction ratio (δ*) are shown to have profound effects on the mixing efficiency 500 μm upstream (*η*_*u*_) and 500 μm downstream (*η*_*d*_) of the island. Islands having small α (e.g., configurations 1 and 4) demonstrate more efficient mixing downstream of the island (i.e. *η*_*d*_ > *η*_*u*_). On the other hand, islands having high α (e.g., configurations 20 and 22) demonstrate more efficient mixing upstream of the island (i.e. *η*_*u*_ > *η*_*d*_). For t = 1 s (right column), homogeneous mixing is more evident, where similar upstream and downstream mixing efficiencies are demonstrated. Superhydrophobic islands are outlined with a red dashed line in each t = 0.4 s image for visualization purposes.

To fully understand the influence of the area and constriction ratios on mixing, the upstream (*η*_*u*_ at *x* = *x*_*u*_) and downstream (*η*_*d*_ at *x* = *x*_*d*_) mixing efficiencies were evaluated for each SDSM configuration at different times (t = 0.4 s and t = 1 s). In certain cases, (*e*.*g*., in configuration 4, Table 1) mixing was better upstream of the island, while in other cases (*e*.*g*., in configuration 22), mixing was better downstream. Seemingly inconclusive, this can be explained by examining the transient mixing behavior near the superhydrophobic island of certain SDSM configurations. Figure 4 shows the transient mixing with various islands for given constriction and area ratios. At t = 0.4 s (left column), for the same constriction ratio (δ* = 0.4), an island with a low area ratio (configuration 1, *α* = 0.02) leads to more efficient mixing downstream (*η*_*d*_ = 0.92) of the island rather than upstream (*η*_*u*_ = *0*.*86*). If the island has a high area ratio, like in configuration 20 (where *α* = 0.06), the liquid cannot flow as easily around the island, thus accumulating in front of the dry island, leading to higher mixing efficiency upstream of the island (*η*_*u*_ = 0.86 vs. *η*_*d*_ = 0.82). Similarly, for an island with very high constriction (δ* = 0.6), a low area ratio made it easier for the liquid to flow around (or over) the island, producing transversal vortices downstream of the obstacle. This leads to higher mixing efficiency downstream of the island than upstream (configuration 4, *η*_*d*_ = 0.84 vs. *η*_*u*_ = 0.67). Conversely, for the same high constriction ratio (δ* = 0.6), an island having a high area ratio (*e*.*g*., configuration 22) promotes mixing via liquid accumulation upstream of the island as the liquid cannot flow as easily around it. In this case, the mixing efficiency was higher upstream (*η*_*u*_ = 0.92) than downstream (*η*_*d*_ = 0.55).

As seen in Fig. 4 for t = 1 s (right column), for configurations 1, 20 and 22, the upstream and downstream mixing efficiencies have stabilized, indicating homogeneous mixing near the superhydrophobic island. This type of mixing behavior is not seen for configuration 4 (*α* = 0.02, δ* = 0.6), where the upstream mixing efficiency (*η*_*u*_ = 0.67) remains much lower than the downstream value (*η*_*d*_ = 0.89). A closer look at the mixing behavior for each of the configurations in Fig. 4 shows that the superhydrophobic islands of the better-performing configurations 1, 20 and 22 remained uncovered (dry) throughout the mixing process, whereas the smaller island for configuration 4 was overcome early with liquid. This indicates that the values chosen for the area ratio and constriction ratio not only influence the upstream and downstream mixing efficiencies, but can also be correlated with the island’s final condition (*i*.*e*. covered or uncovered) after advective transport over the *C*_*Wedge*_ domain. As the two liquids are transported in the *C*_*Wedge*_ and approach the superhydrophobic island, the size of the island and the effective width (*i*.*e*. *δ*_w_ (*x*) − *δ*_*i*_ (*x*)) of the superhydrophilic regions adjacent to the island through which the liquid can pass, ultimately determine if the island would remain uncovered, or be inundated with liquid. Larger islands (high *α*) with adequate paths on either side of the island (implying low values of δ*) prevent liquid from inundating the island. However, for the opposite case of low area ratio but high constriction ratio, the superhydrophobic island was covered with the liquid mixture (there was not enough space on either side of the island for the fluid to pass). It is important to note that for special cases (*e*.*g*., configurations 1, 7, 9, *etc*.), some SDSM displayed instances in which the island was covered, and other instances in which the island remained uncovered over the course of multiple trials (see Supporting Videos SM2 and SM3 for configuration 12). This ambivalence indicates the marginal conditions where island wetting occurs and is explained in more detail later in the paper.

**Figure 5:**
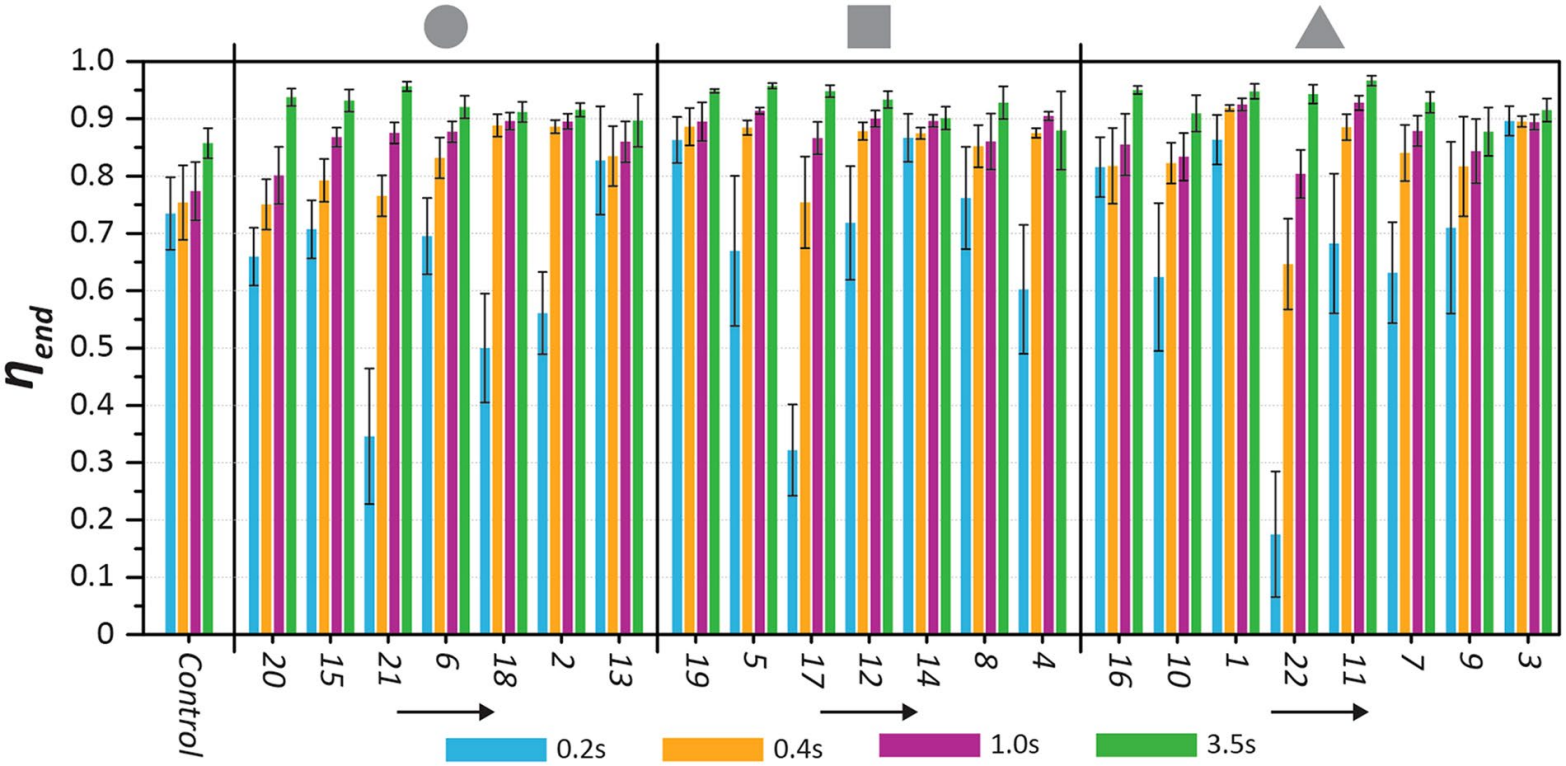
Variation of mixing efficiency (*η*_*end*_) with time at the end of the C_Wedge_ domain for each SDSM configuration (see Table 1). SDSM were grouped first according to the respective shape of the superhydrophobic island (denoted at top), and then with increasing probability of island inundation in each group (indicated by black arrows beneath each shape group).

Figure 5 presents a performance map of the entire SDSM design (Table 1) and the control case micromixer: it also demonstrates how the island inundation by liquid can influence mixing at the end (*x*_*end*_) of the *C*_*Wedge*_ domain. Here, all SDSM configurations are grouped first according to the shape of the island (circle, square or triangle, separated by vertical black lines) and further sub-grouped according to increasing probability of island inundation by liquid (left to right in each of three shape sets). In cases where the superhydrophobic island had a high probability of staying dry, mixing was generally more efficient (*η*_*end*_ ~ 0.95 at *t* = 3.5 s). For cases where the island was inundated with liquid, mixing was faster (*η*_*end*_ ~ 0.85 for *t* = 0.4 s), albeit leading to a slight decrease in overall mixing efficiency (*e*.*g*., *η*_*end*_ at *t* = 3.5 s is ~0.9, 0.88, and 0.92, respectively, for the circular, rectangular and triangular islands for the highest coverage probability cases). In general, liquid mixing reached efficiency over 0.8 within 1 s. The mixing efficiency for the control case increased from ~0.73 at 0.2 s to ~0.77 at 1 s, while beyond *t* = 3.5 s, the mixing efficiency stabilized at ~0.86 (well below the values attained by the island-including SDSM). Out of the cases presented in Fig. 5, configuration 1 appears to yield the fastest mixing with a high mixing efficiency (*η*_*end*_ ~ 0.92 within 0.4 s). As also seen from the mixing images in Figs 2 and 4, and in the Supplementary Video SM1, capillary-driven advection persists for the first 250 ms of mixing (on average), after which molecular diffusion becomes dominant. From this observation, depending on the mixing performance parameter (*i*.*e*. mixing efficiency or mixing time), the design of the present SDSM can be tuned to cater to a specific mixing task for a typical surface microfluidic platform.

**Figure 6:**
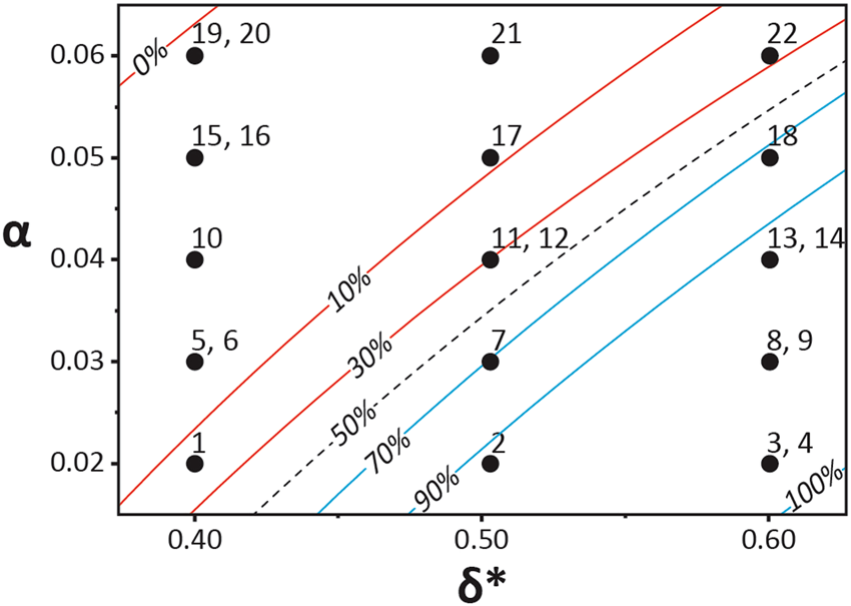
Probability of coverage for a superhydrophobic island (calculated with JMP® statistical software) as a function of area ratio (*α*) and constriction ratio (δ*). Each iso-curve represents the probability for an island with a given area ratio and given constriction ratio to be covered with the oncoming liquid. Red curves indicate a lower probability for an island to be covered by liquid, whereas the blue curves indicate a higher chance of the island to be covered with liquid. If any particular island has area ratio and constriction values which lie between two iso-probability lines, the probability that the island will be covered lies between the two probability values of the adjoining curves.

As mentioned earlier, the area and constriction ratios of the superhydrophobic island on a SDSM can be correlated to the island’s wet or dry condition after the mixing process. This correlation, mapped quantitatively in Fig. 6, offers insight in SDSM design to achieve a target mixing performance level. Here, the wet-dry condition of the island for *each mixing experiment* (five trials for each SDSM configuration) has been included to show the extent of island coverage in the form of iso-probability lines overlaid onto the α-δ* plot. The 100% probability line (lower right corner of the plot) indicates that an island will almost certainly be covered with liquid during the mixing process. By selecting certain values of the area and constriction ratios for a given superhydrophobic island, as per Fig. 6, one can specifically tune the probability that the island becomes inundated or remains dry during the mixing process. This, in turn, can ultimately influence the mixing performance of the SDSM. For example, SDSM designs near the 0% coverage probability line (*e*.*g*. configurations 19, 20, both having high area ratio and low constriction ratio) facilitate higher long-term and lower short-term mixing efficiencies (see Fig. 5). Conversely, SDSM designs near the 100% coverage probability line (*e*.*g*., configurations 3, 4, having low area ratio but high constriction ratio) facilitate higher short-term (<1 s) but moderate long-term mixing efficiencies. Therefore, when ultimate mixing efficiency is prioritized at the expense of rapid mixing, then a SDSM near the 0% coverage probability line in Fig. 6 should be prescribed. It is important to note that when designing a SDSM with an island of certain area and constriction ratio values, the size of the *C*_*Wedge*_ must also be taken into consideration. There are limits to the values chosen for these island parameters, meaning that a particular island with extreme geometries may not fit within *C*_*Wedge*_. Since the axial location (*x*) of the island depends on the area ratio and constriction ratio, certain values chosen for these two island parameters may suggest an axial location that falls outside the design constraints of *C*_*Wedge*_.

## Conclusions

We have demonstrated pumpless transport and mixing of two liquid microvolumes dispensed on a self-driven surface micromixer (SDSM) fabricated by spray casting a TiO_2_-fluoropolymer superhydrophobic composite coating onto a PET substrate and then selectively treating certain areas with UV radiation to induce local wettability. The fabrication process is facile and scalable, and can easily be extended to other types of substrates (paper, glass, *etc*.). The presence of a separate mixing and detection zone (*C*_*Wedge*_) is a unique feature of the approach. The SDSM harnesses surface tension forces with three strategically-patterned superhydrophilic wedge-shaped tracks on a superhydrophobic background, which effectively mixed two aqueous reacting liquids without external energy input. The two liquids were dispensed drop-wise at the narrow ends of the two juxtaposed, superhydrophilic tracks (A and B); liquid droplets experienced capillary-driven transport to the wider end of the tracks, where they formed adjacent liquid bulges. Upon dispensing beyond a threshold volume, the two bulges coalesced. The merged liquid was then transported by a Laplace pressure gradient along a third superhydrophilic track (*C*_*Wedge*_) where liquid mixing continued. Strategically-placed, planar, superhydrophobic islands of various shapes, sizes and orientations were also patterned on *C*_*Wedge*_ to alter the flow field in a way intended to affect mixing. The extent of liquid mixing on various SDSM designs was quantified through transmitted light intensity analysis. Although the introduction of superhydrophobic islands lowered the early mixing efficiency, the long-term mixing improved (highest mixing efficiency of 0.97 was observed after 3.5 s, as opposed to 0.86 for the control case). It was found that either the mixing time or mixing efficiency could be tuned by allowing the liquid to cover the island during mixing or to simply flow around the island; this was achieved by changing the constriction and area metrics of the island. In general, efficient mixing improved at the expense of rapid mixing when using an island design with a high area ratio and low constriction ratio. Conversely, rapid –but not very efficient- mixing, was attained by a combination of low area and high constriction ratios. The mixing capability of this novel design will be helpful in designing point of care diagnostics devices, lab-on-a-chip applications, and other surface microfluidic platforms where liquid microvolume mixing must occur in sub-second time frames.

## Materials, Methods and Characterization

### SDSM Fabrication

**Figure 7:**
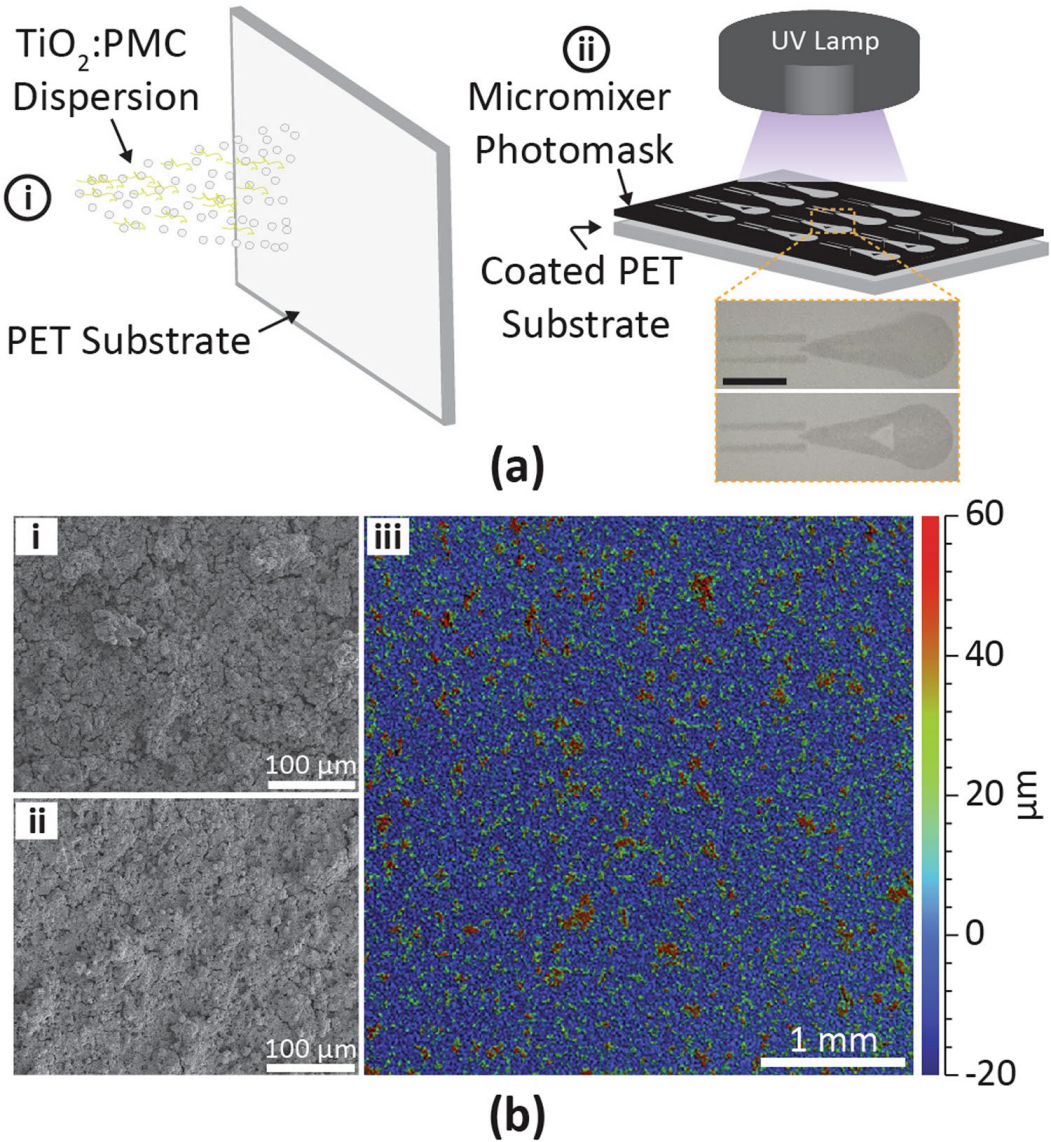
(**a**) Fabrication steps of self-driven surface micromixers (SDSM): (i) TiO_2_:PMC dispersions are sprayed onto the PET substrate. (ii) The superhydrophobic TiO_2_:PMC-coated substrates received partial UV treatment using a photomask. The inset shows top-view images of an actual SDSM: (top) control case with no island, and (bottom) SDSM with triangular, superhydrophobic island. The black scale bar in the top image of the inset denotes 1 cm and applies to both photos. (**b**) Despite the wettability contrast of the superhydrophobic island and superhydrophilic surroundings, the morphology and topography of these two regions are consistent. SEM images of the (i) superhydrophobic, and (ii) superhydrophilic regions of the TiO_2_:PMC surface. (iii) Surface height profile of a domain of a SDSM encompassing a square, superhydrophobic island which cannot be distinguished in this profile map (meaning that UV treatment does not modify the physical texture of the SDSM surface).

The fabrication process of the SDSM, as demonstrated schematically in Fig. 7a, consists of several simple and scalable steps. For a typical batch, 1 g of titanium (IV) oxide (TiO_2_) nanoparticles (<25 nm, Anatase; Sigma-Aldrich) was dispersed in 13.25 g of ethanol (200 proof; Decan Labs). The dispersion was hand-shaken and subsequently probe-sonicated with 1000 J energy (750 W, 13 mm probe dia., 40% amplitude; Sonics & Materials, Inc., Model VCX-750). Immediately after sonication, 1.25 g of a perfluoroalkyl methacrylate copolymer (PMC) (20 wt.% in water; DuPont, Capstone ST-100) was added to the TiO_2_-ethanol mixture to ultimately formulate a TiO_2_:PMC dispersion (particle filler to polymer mass fraction ~ 80%). The dispersion was left to stabilize for approximately 24 hours, allowing the PMC to swell in the ethanol dispersion. After stabilizing, the dispersion was placed in an ultrasonic bath (8891 Ultrasonic Cleaner, 2.5 gallon; Cole-Parmer) for approximately 10 minutes, and then sprayed onto 75 × 115 mm sheets of polyethylene terephthalate (PET) (PP2500, 3 M). Spraying was carried out using a siphon feed airbrush (0.73 mm nozzle, 205 kPa; VL-Set, Paasche), and the Polyethylene Terephthalate (PET) substrates were sprayed from a distance of 25 cm to ensure a uniform TiO_2_:PMC nanocomposite coating (see Fig. 7a-i). Following the spray step, the substrates were heat-treated in a convection oven (Model 10GC; Quincy Lab, Inc.) at 80 °C for 2 hours. As the PMC is intrinsically hydrophobic (a smooth PMC film has an apparent contact angle *θ* ^*^ ~ 117° for water)^52^, the addition of TiO_2_ provides adequate hierarchical (micro- to nano-scale) roughness, rendering the final coating superhydrophobic $({\theta }_{water}^{\ast } \sim 160^\circ \pm 2^\circ )$. Another unique aspect of the TiO_2_: PMC coatings, attributed to the photocatalytic nature of Anatase TiO_2_, is that upon UV exposure, the wettability of the coating can be easily converted from very low (superhydrophobic behavior) to very high (superhydrophilic behavior)^53–56^.

The SDSM was fabricated by selectively exposing the superhydrophobic TiO_2_:PMC substrates to UV irradiation for 30 minutes under a 400 W UV lamp (390 nm, Dymax™ 5000 EC) through a PET photomask printed in a common laserjet printer. Each photomask allows UV light to enter through any unprinted (transparent) regions, initiating a photocatalytic reaction that rendered the *UV-exposed* regions superhydrophilic while retaining the superhydrophobic properties of the *unexposed* regions (see Fig. 7a-ii). The superhydrophilic and superhydrophobic regions of the SDSM were characterized using a Hitachi S-3000N scanning electron microscope (SEM), in conjunction with a Bruker Contour GT-K optical profilometer. The absence of any noticeable difference in morphology and topography between the superhydrophilic and superhydrophobic regions from the SEM and profilometry images (Fig. 7b) suggests that the difference in wettability between these regions is attributed to the chemical change of PMC within the TiO_2_:PMC coatings during UV treatment.

### Island Parameters

The mixing performance of the SDSM was investigated over various design parameters, *e*.*g*., island shapes (circle, square, triangle); area ratios (0.03, 0.04, 0.05, 0.06, see Eq. 1); constriction ratios (0.4, 0.5, 0.6, see Eq. 2) and orientations (0, 45, 90, 180°). The values of the area ratio (*α*) and the constriction ratio (δ*) were chosen based on two criteria; first to ensure that the island fit within *C*_*Wedge*_, and second, to avoid an island severely restricting transport along *C*_*Wedge*_. The axial distance where the island was placed (*x*_*i*_) varied with *α*, δ* and the shape of the island (see Fig. 1a). For example, a circular island with *δ*_*i*_ ~2.1 mm, *α* = 0.03 and δ* = 0.4 had its center placed at *x*_*i*_ = 8.43 mm, so that the local wedge width (*δ*_*w*_ ~5.21 mm) was sufficiently large to allow liquid flow around the island. Since endless combinations of SDSM configurations were possible with the given design parameters, a design of experiments (DOE) software (JMP®, Version 12, SAS Institute Inc., Cary NC, 1989–2007) was used to construct the experimental parameter matrix.

### Mixing Efficiency Calculations

The liquid mixing efficiency (*i*.*e*. degree of mixing homogeneity) was quantified as a function of time, using a pixel color intensity analysis at various locations (*x*_*u*_, *x*_*d*_, and *x*_*end*_; for details refer to Figure S4 of the SI) of the SDSM. For each analysis location (*x*) and time (index *k*), quantification involved several steps of calculation. First, the intensity (*I*_*j*,*k*_) of a *j* ^*th*^ pixel along the analysis station for a given *k* ^*th*^ time was normalized with the intensity of the same pixel prior to (*I*_*j*,0_) and after mixing (*I*_*j*,∞_) as

4 $${I}_{j,k}^{\ast }=|\frac{{I}_{j,k}-{I}_{j,0}}{{I}_{j,\infty }-{I}_{j,0}}|.$$

The normalized pixel intensity values $({I}_{j,k}^{\ast })$ were then averaged along the width of the analysis station to yield ${\bar{I}}_{k}$, the average normalized pixel intensity for a given *k* ^*th*^ time and location (*x*)

5 $${\bar{I}}_{k}=\sum _{j=1}^{{N}_{\delta }}\frac{{I}_{j,k}^{\ast }}{{N}_{\delta }},$$

where *N*_*δ*_ is the number of pixels along the track width (*δ*_*w*_). The degree of mixing homogeneity (*σ*_*k*_) and the mixing efficiency (*η*_*k*_) for a given location (*x*) and time (*k*) was ultimately calculated by

6 $$\begin{array}{cc}{\sigma }_{k}=\sqrt{\frac{1}{{N}_{\delta }}\sum _{j=1}^{{N}_{\delta }}{(\frac{{I}_{j,k}^{\ast }-{\bar{I}}_{k}}{{\bar{I}}_{k}})}^{2}}, & {\rm{and}}\end{array}$$

7 $${\eta }_{k}=1-{\sigma }_{k}.$$

According to the above definitions, the values of *η* would range from 0 to 1 – unity denoting perfect mixing, and zero implying no mixing. The rationale of calculating the mixing efficiency is provided in the SI (Section S5 and Fig. S4).

## Electronic supplementary material

SM1-Control

SM2-CoveredIsland

SM3-UncoveredIsland

Supplementary Information

## References

[1] Chin CD, Linder V, Sia SK (2012). Commercialization of microfluidic point-of-care diagnostic devices. Lab Chip.

[2] Chin CD (2011). Microfluidics-based diagnostics of infectious diseases in the developing world. Nat. Med..

[3] Haeberle S, Zengerle R (2007). Microfluidic platforms for lab-on-a-chip applications. Lab Chip.

[4] Weigl BH, Bardell RL, Cabrera CR (2003). Lab-on-a-chip for drug development. Advanced Drug Delivery Reviews.

[5] Whitesides GM (2006). The origins and the future of microfluidics. Nature.

[6] West J, Becker M, Tombrink S, Manz A (2008). Micro total analysis systems: latest achievements. Anal. Chem..

[7] Seemann R, Brinkmann M, Pfohl T, Herminghaus S (2012). Droplet based microfluidics. Rep. Prog. Phys..

[8] Tice JD, Song H, Lyon AD, Ismagilov RF (2003). Formation of droplets and mixing in multiphase microfluidics at low values of the Reynolds and the capillary numbers. Langmuir.

[9] Washizu M (1998). Electrostatic actuation of liquid droplets for microreactor applications. IEEE Transactions on Industry Applications.

[10] Shembekar N, Chaipan C, Utharala R, Merten CA (2016). Droplet-based microfluidics in drug discovery, transcriptomics and high-throughput molecular genetics. Lab Chip.

[11] Davanlou A, Kumar R (2015). Passive mixing enhancement of microliter droplets in a thermocapillary environment. Microfluidics and Nanofluidics.

[12] Xing S, Harake RS, Pan T (2011). Droplet-driven transports on superhydrophobic-patterned surface microfluidics. Lab Chip.

[13] Balu B, Berry AD, Hess DW, Breedveld V (2009). Patterning of superhydrophobic paper to control the mobility of micro-liter drops for two-dimensional lab-on-paper applications. Lab Chip.

[14] Göröcs Z (2013). Giga-pixel fluorescent imaging over an ultra-large field-of-view using a flatbed scanner. Lab Chip.

[15] Lehmann M (2015). On-chip recalcification of citrated whole blood using a microfluidic herringbone mixer. Biomicrofluidics.

[16] Yetisen AK, Akram MS, Lowe CR (2013). Paper-based microfluidic point-of-care diagnostic devices. Lab Chip.

[17] Cho SK, Moon HJ, Kim CJ (2003). Creating, transporting, cutting, and merging liquid droplets by electrowetting-based actuation for digital microfluidic circuits. Journal of Microelectromechanical Systems.

[18] Lu LH, Ryu KS, Liu C (2002). A magnetic microstirrer and array for microfluidic mixing. Journal of Microelectromechanical Systems.

[19] Roy, T., Sinha, A., Chakraborty, S., Ganguly, R. & Puri, I. K. Magnetic microsphere-based mixers for microdroplets. *Phys*. *Fluids* **21**, doi:10.1063/1.3072602 (2009).

[20] Jones TB, Gunji M, Washizu M, Feldman MJ (2001). Dielectrophoretic liquid actuation and nanodroplet formation. J. Appl. Phys..

[21] Guttenberg Z (2005). Planar chip device for PCR and hybridization with surface acoustic wave pump. Lab Chip.

[22] Martinez AW, Phillips ST, Whitesides GM, Carrilho E (2010). Diagnostics for the developing world: microfluidic paper-based analytical devices. Anal. Chem..

[23] Osborn JL (2010). Microfluidics without pumps: reinventing the T-sensor and H-filter in paper networks. Lab Chip.

[24] Ju J, Warrick J (2013). Passive micromixer using by convection and surface tension effects with air-liquid interface. Biochip J.

[25] Yang ID, Chen YF, Tseng FG, Hsu HT, Chieng CC (2006). Surface tension driven and 3-D vortex enhanced rapid mixing microchamber. Journal of Microelectromechanical Systems.

[26] Yeh SI, Fang WF, Sheen HJ, Yang JT (2013). Droplets coalescence and mixing with identical and distinct surface tension on a wettability gradient surface. Microfluidics and Nanofluidics.

[27] Chung CK, Lai CC, Shih TR, Chang EC, Chen SW (2013). Simulation and fabrication of capillary-driven meander micromixer for short-distance mixing. Micro Nano Lett.

[28] Swickrath MJ, Burns SD, Wnek GE (2009). Modulating passive micromixing in 2-D microfluidic devices via discontinuities in surface energy. Sens. Actuators, B.

[29] Delaney JL, Hogan CF, Tian J, Shen W (2011). Electrogenerated chemiluminescence detection in paper-based microfluidic sensors. Anal. Chem..

[30] Nilghaz A, Shen W (2015). Low-cost blood plasma separation method using salt functionalized paper. RSC Adv.

[31] Yamada K, Takaki S, Komuro N, Suzuki K, Citterio D (2014). An antibody-free microfluidic paper-based analytical device for the determination of tear fluid lactoferrin by fluorescence sensitization of Tb3+. Analyst.

[32] Bhakta SA, Borba R, Taba M, Garcia CD, Carrilho E (2014). Determination of nitrite in saliva using microfluidic paper-based analytical devices. Anal. Chim. Acta.

[33] Krüger J (2002). Development of a microfluidic device for fluorescence activated cell sorting. Journal of Micromechanics and Microengineering.

[34] Nie Z (2010). Electrochemical sensing in paper-based microfluidic devices. Lab Chip.

[35] Alheshibri MH, Rogers NG, Sommers AD, Eid KF (2013). Spontaneous movement of water droplets on patterned Cu and Al surfaces with wedge-shaped gradients. Appl. Phys. Lett..

[36] Ghosh A, Ganguly R, Schutzius TM, Megaridis CM (2014). Wettability patterning for high-rate, pumpless fluid transport on open, non-planar microfluidic platforms. Lab Chip.

[37] Hong LF, Pan TR (2011). Surface microfluidics fabricated by photopatternable superhydrophobic nanocomposite. Microfluidics and Nanofluidics.

[38] Khoo, H. S. & Tseng, F. G. Spontaneous high-speed transport of subnanoliter water droplet on gradient nanotextured surfaces. *Appl*. *Phys*. *Lett*. **95**, 063108, doi:10.1063/1.3197574 (2009).

[39] Schutzius TM, Elsharkawy M, Tiwari MK, Megaridis CM (2012). Surface tension confined (STC) tracks for capillary-driven transport of low surface tension liquids. Lab Chip.

[40] Alam A, Afzal A, Kim KY (2014). Mixing performance of a planar micromixer with circular obstructions in a curved microchannel. Chem. Eng. Res. Des..

[41] Nguyen TNT, Kim MC, Park JS, Lee NE (2008). An effective passive microfluidic mixer utilizing chaotic advection. Sens. Actuators, B.

[42] Seo HS, Kim YJ (2012). A study on the mixing characteristics in a hybrid type microchannel with various obstacle configurations. Mater. Res. Bull..

[43] Wang H, Iovenitti P, Harvey E, Masood S (2002). Optimizing layout of obstacles for enhanced mixing in microchannels. Smart Mater. Struct..

[44] Boonyasit Y, Laiwattanapaisal W (2015). A microfluidic paper-based analytical device for the assay of albumin-corrected fructosamine values from whole blood samples. Bioanalysis.

[45] Lin SC (2016). Paper-based CRP Monitoring Devices. Sci. Rep..

[46] Yang XX, Forouzan O, Brown TP, Shevkoplyas SS (2012). Integrated separation of blood plasma from whole blood for microfluidic paper-based analytical devices. Lab Chip.

[47] Eiswirth RT, Bart HJ, Ganguli AA, Kenig EY (2012). Experimental and numerical investigation of binary coalescence: Liquid bridge building and internal flow fields. Phys. Fluids.

[48] de Gennes, P.-G., Brochard-Wyart, F. & Quéré, D. *Capillarity and wetting phenomena: drops*, *bubbles*, *pearls*, *waves*. (Springer Science & Business Media, 2004).

[49] Peters CA, French CL (1941). Study of Ferric Thiocyanate Reaction. Ind. Eng. Chem.

[50] Hessel V, Hardt S, Löwe H, Schönfeld F (2003). Laminar mixing in different interdigital micromixers: I. Experimental characterization. AlChE J.

[51] Antony, J. Design of Experiments for Engineers and Scientists. (Elsevier Ltd, 2003).

[52] Mates JE, Schutzius TM, Qin J, Waldroup DE, Megaridis CM (2014). The Fluid Diode: Tunable Unidirectional Flow through Porous Substrates. Acs Appl. Mater. Inter..

[53] Diebold U (2003). The surface science of titanium dioxide. Surf. Sci. Rep..

[54] Fujishima A, Rao TN, Tryk DA (2000). Titanium dioxide photocatalysis. Journal of Photochemistry and Photobiology C: Photochemistry Reviews.

[55] Xu QF, Liu Y, Lin FJ, Mondal B, Lyons AM (2013). Superhydrophobic TiO2-polymer nanocomposite surface with UV-induced reversible wettability and self-cleaning properties. Acs Appl. Mater. Inter..

[56] Yang M, Di Z, Lee JK (2012). Facile control of surface wettability in TiO2/poly(methyl methacrylate) composite films. J. Colloid Interface Sci..

