# Supplementary material for: Rapid, Self-driven Liquid Mixing on Open-Surface Microfluidic Platforms
Source: Sci Rep. 2017 May 11;7:1800. doi: 10.1038/s41598-017-01725-0 (PMC5431963; doi:10.1038/s41598-017-01725-0)
Supplement: Supplementary file 4 — Supplementary Information [file 41598_2017_1725_MOESM4_ESM.pdf]

Supplementary Information

Rapid, Self-driven Liquid Mixing on Open-Surface Microfluidic Platforms

Jared M. Morrissette,[1] Pallab Sinha Mahapatra,[1] Aritra Ghosh,[1] Ranjan Ganguly[2] and Constantine M. Megaridis[1*]

[1] Department of Mechanical and Industrial Engineering, University of Illinois at Chicago, Chicago, IL 60607, United States

[2] Department of Power Engineering, Jadavpur University, Kolkata 700098, India

[*] Corresponding author: E-mail address cmm@uic.edu

Supplementary Videos

- *SM1-Control*: Video demonstrating each step of a typical mixing process (*e.g.* coalescence, transport, *etc.*) for the base case (control) SDSM
- *SM2-CoveredIsland*: Demonstration of mixing on an SDSM (configuration 12) in which the superhydrophobic island was inundated with liquid
- *SM3-UncoveredIsland*: Demonstration of mixing on an SDSM (also configuration 12) in which the superhydrophobic island remained dry

S1: Definition of δ_i for various island shapes and orientations (θ)

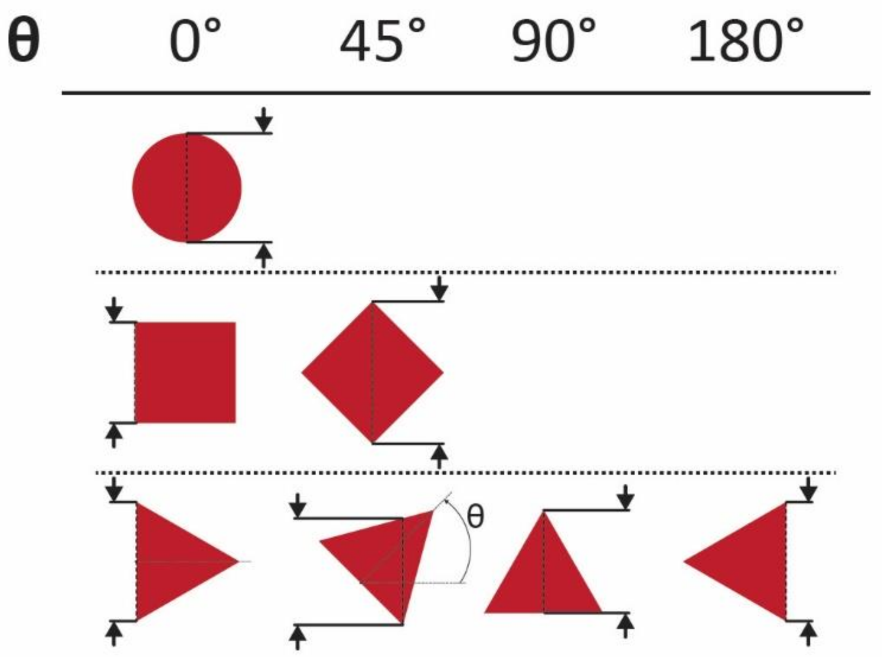

Figure S1: *Definition of the width of a superhydrophobic island (δ_i – dashed lines) for various island shapes and orientations. The value of δ_i for a given island shape and orientation defines the axial location (x_i) where this measurement is done (see Figure 1a of the manuscript). In general, δ_i was at the widest part of the island, except for a triangular shaped island rotated 45°.*

S2: Transient dynamics of the liquid front on the C-Track for the *Control Case*

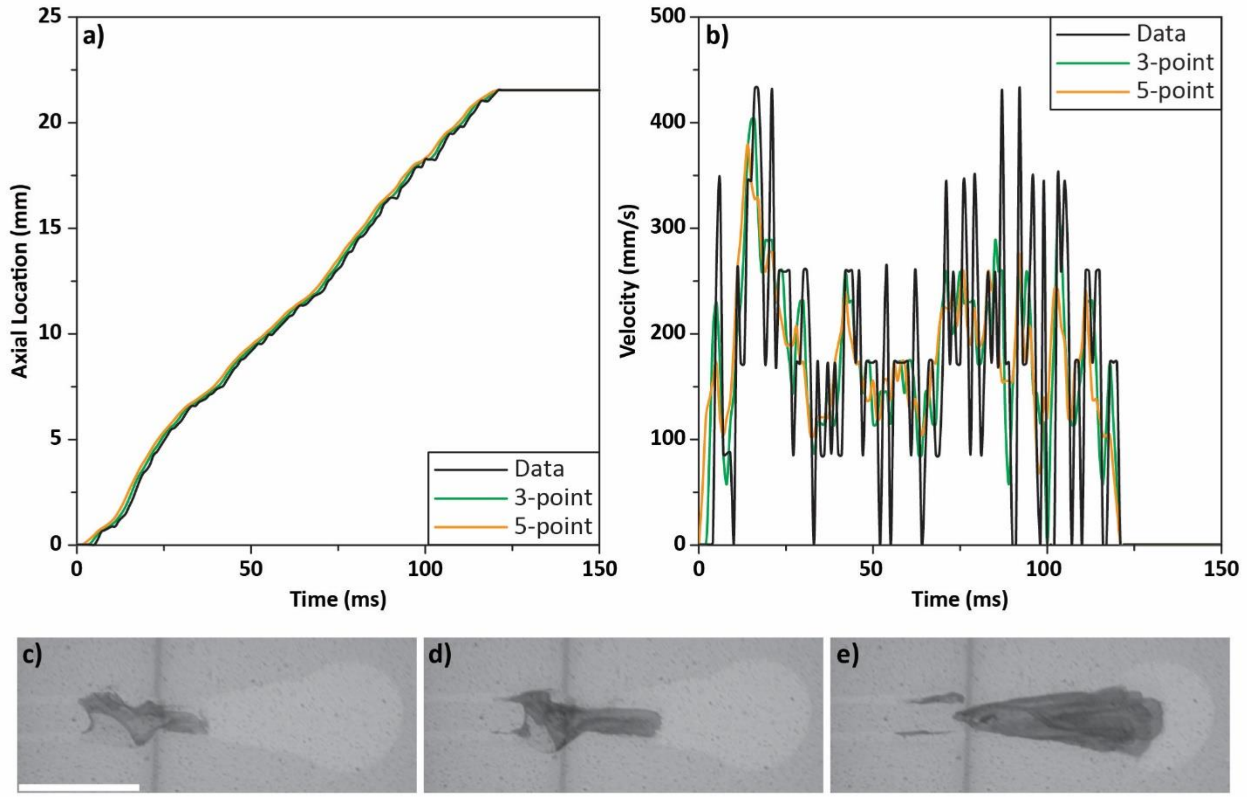

Figure S2: ***(a)*** *Displacement (axial location) of the liquid front on the C-track during advective transport on an SDSM without a superhydrophobic island (Control Case). Each data point corresponds to the axial location of the liquid front during propagation onto the C-track and was manually tracked from the images of the mixing events (every 1 ms).* ***(b)*** *Velocity of the liquid front during propagation, as calculated from the displacement data. For both (a) and (b), the black line represents the raw data. To reduce noise, 3-point and 5-point moving point averaging was applied to the raw data and each is represented by solid green and orange lines, respectively.* ***(c-e)*** *Images corresponding to the location of the liquid front during advective transport on the* C_{wedge} *at (c) 25 ms, (d) 50 ms, and (e) 100 ms. The scale bar in (c) marks 1 cm, and also applies to (d) and (e).*

S3: 4.7 µL droplets of NH_4SCN and $FeCl_3$ on the superhydrophobic background of a SDSM

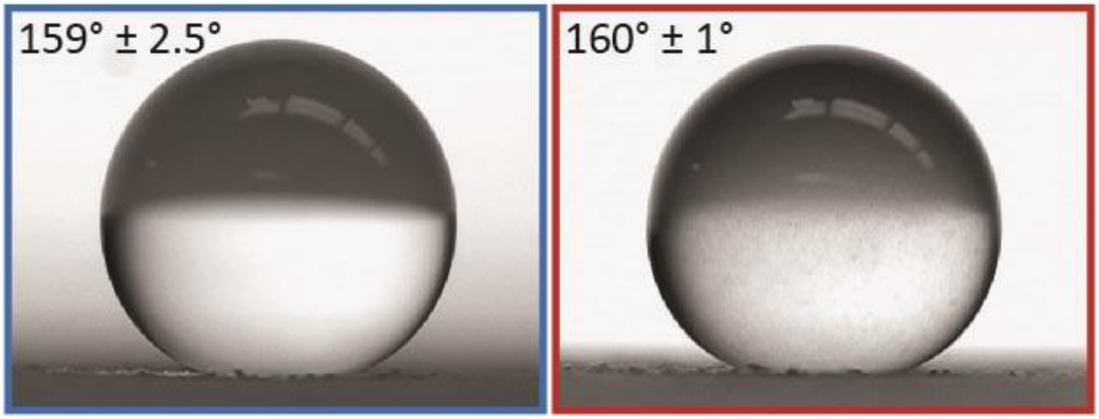

Figure S3: *Apparent contact angle images of the two probe solutions which were dispensed onto the superhydrophobic region of the SDSM:* ***(Left)*** *0.5M ammonium thiocyanate (NH_4SCN) in H_2O, and* ***(Right)*** *0.25M ferric chloride ($FeCl_3$) in H_2O. Both solutions are aqueous, so their wetting behavior is similar to pure water.*

S4: Explanation of interaction plots (*i.e.,* Figure 3 of the manuscript)

In a parametric study involving multiple variables, an interaction plot displays the extent to which the effect of one factor (independent variable) on the response variable (the dependent variable) changes depending on the level of the other factor(s). For example, a 2-dimensional interaction plot would explain whether the variation of a dependent variable z(*x*,*y*) – where *x* and *y* are the independent variables – with *x* (or *y*) is influenced by values of the other variable *y* (or *x*) at which the *z* data are reported. The lines in each subplot in ***Figure 3*** of the manuscript were generated using a least squares regression analysis built in the DOE software (JMP, SAS®). In general, for a set of data points $\{(x, y, z): x \in X, y \in Y, z \in Z\}$, with one dependent variable (*z*) and two independent variables (*x* and *y*), a linear expression, considering a first-order interaction between *x* and *y* can be derived and fit to the dataset using the following expression (similar to the method outlined by Jaccard et al.[1])

$$z = b_0 + b_1 x + b_2 y + b_3 xy + e \qquad \textbf{\textit{(E1)}}$$

Eq. E1, when rearranged, becomes

$$z = (b_0 + b_2 y) + x(b_1 + b_3 y) + e \qquad \textbf{\textit{(E2)}}$$

The first and second parenthesis terms of ***Eq. E2*** represent the intercept and slope of the *z(x)* line, respectively, and *e* denotes a residual term, which is essentially the difference between the actual values of *z* (*i.e.* from the data) and the expected values of *z* (*i.e.* from least-squares fitting). The coefficients b_0, b_1, b_2, b_3 and *e* are calculated during the least-squares regression analysis,[2] and depending on their values, it is possible to determine if there is a first-order interaction between the independent variables *x* and *z*.

To demonstrate a relevant example of how a first-order interaction between two independent variables can influence a dependent variable, let us consider a simple case where the area ratio (α) and constriction ratio (δ^*) are the *only* independent variables that can influence the mixing efficiency (η); in doing so, we neglect any influences from the shape or orientation (θ) of the superhydrophobic island. By plotting η against α for a constant value of δ^*, one can write ***Eq. E2*** as

$$\eta = (b_0 + b_2 \delta^*) + \alpha(b_1 + b_3 \delta^*) + e \qquad \textbf{\textit{(E3)}}$$

To fully understand how η is influenced by the interaction between α and $\delta*$, let us choose two constant values for $\delta*$, a low value and a high value, δ_l^* and δ_h^*, respectively. The two lines are then represented by the following equations

$$\eta_l = (b_0 + b_2\delta_l^*) + \alpha(b_1 + b_3\delta_l^*) + e \quad \textbf{\textit{(E4)}}$$

$$\eta_h = (b_0 + b_2\delta_h^*) + \alpha(b_1 + b_3\delta_h^*) + e \quad \textbf{\textit{(E5)}}$$

The difference in slopes of these two lines is $[b_3(\delta_h^* - \delta_l^*)]$. If there is no interaction between α and $\delta*$, $b_3 = 0$ and the two curves are parallel with slopes equal to b_1 and have a y-intercept offset of $b_2(\delta_h^* - \delta_l^*)$. However, if there are interactions present, $b_3 \neq 0$; hence the slopes of η_l $(= b_1 + b_3\delta_l^*)$ and η_h $(= \ b_1 + b_3\delta_h^*)$ differ.

It is important to note that the above example only considers first-order interactions between *two* independent variables. A least-squares regression analysis, which considers interactions between three or more independent variables, is more complicated to describe here. ***Figure 3*** in the manuscript considers that there are *first-order* interactions between *four* independent variables (α, $\delta*$, shape, and θ). A general form describing how η varies with α, while considering interactions between α, $\delta*$, shape, and θ may be written as:

$$\eta = m\alpha + B + e \ , \quad \textbf{\textit{(E6)}}$$

where the slope (m) and η-intercept (B) are given by

$$m = b_0 + b_2\delta^* + b_3(shape) + b_4\theta + b_8(shape)\delta^* + b_9\theta\delta^* + b_{10}\theta(shape) \quad \textbf{\textit{(E7)}}$$

$$B = b_1 + b_5\delta^* + b_6(shape) + b_7\theta \quad \textbf{\textit{(E8)}}$$

The least-squares regression analysis for the four independent variables (α, $\delta*$, shape, θ) was performed using the JMP statistical software (SAS®), which generated the sub-plots in ***Figure 3*** of the manuscript. Further details on interactions and regression analysis for three or more independent variables are given in Dawson and Richter[3] and Aiken et al.[4]

S5: Mixing efficiency calculation from image analysis

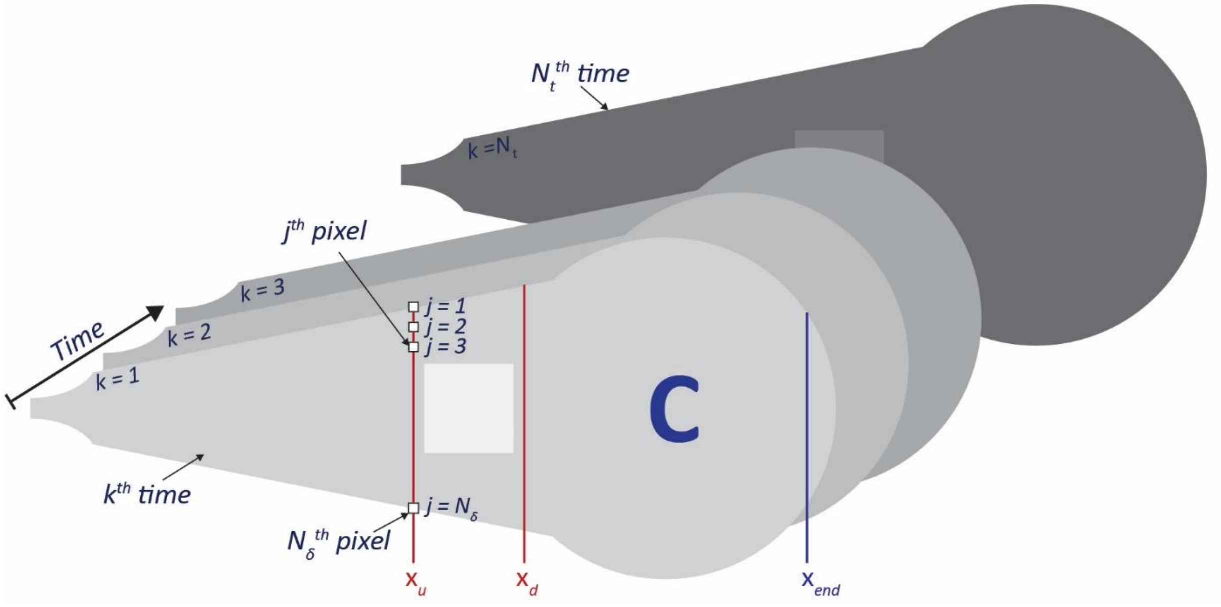

Figure S4: *Superhydrophilic wedge track (grey shape) with a superhydrophobic island (white).* x_{end} *is common for both the control case (no island) and SDSM with a superhydrophobic island, and is approximately 1 mm from the far edge of* $C_{Reservoir}$. x_u *and* x_d *are locations on a SDSM having a superhydrophobic island, and are approximately 500 µm upstream and downstream from the island edges, respectively. A pixel analysis was carried out at each location every millisecond beginning with t = 0 ms, and up to t =* N_t *ms.*

Mixing homogeneity (σ_k) and mixing efficiency (η_k) values were calculated for every millisecond (k^{th} instant of time) of transport on the SDSM. Since the SDSM displayed some non-uniformities in TiO_2 particle distribution, spatial heterogeneity to transmitted light on the substrate was observed even on a dry track. Regions with higher concentrations of coating nanoparticles appeared darker than regions of lower concentrations. Therefore, the mixing homogeneity on a wet track was calculated from the pixel information of a transient image after normalizing the local pixel intensity values with the respective pixel intensity values on the dry substrate. The mixing efficiency at a particular time (k) for a particular location (x_u, x_d, and x_{end}) was calculated using the following algorithm (***Figure S4***):

1. Identify the pixel intensity at a particular pixel point (*j^th^* pixel) before and after complete mixing ($I_{j,0}$ and $I_{j,\infty}$, respectively).
2. Identify the pixel intensity ($I_{j,k}$) at a particular location (*j^th^* pixel) at a particular time (*k^th^* time instant). Normalize each $I_{j,k}$ with respect to $I_{j,0}$and $I_{j,\infty}$ using

$$I_{j,k}^{*} = \left|\frac{I_{j,k}-I_{j,0}}{I_{j,\infty}-I_{j,0}}\right| \quad \textbf{(E9)}$$

3. Calculate the average, normalized intensity value at given *x* (*i.e.* x_u, x_d, or x_{end}) location ($\bar{I}_k$) for all pixel points (N_δ) at a particular section width (δ_w) of C_{Wedge}

$$\bar{I}_k = \sum_{j=1}^{N_\delta} \frac{I_{j,k}^{*}}{N_\delta} \quad \textbf{(E10)}$$

4. Calculate the variance of each jth pixel intensity ($I_{j,k}^{*}$) by normalizing with $\bar{I}_k$ at a given time (k^{th} time) for each *x* considered along the wettable C_{Wedge}

$$\sigma_k = \sqrt{\frac{1}{N_\delta}\sum_{j=1}^{N_\delta}\left(\frac{I_{j,k}^{*}-\bar{I}_k}{\bar{I}_k}\right)^2} \quad \textbf{(E11)}$$

5. Calculate the mixing efficiency at a particular time *k* as

$$\eta_k = 1 - \sigma_k \quad \textbf{(E12)}$$

For a relatively unmixed state, where local intensities along a transverse line at a given axial position varied widely from the average, the above procedure produced large values of σ, and hence, low efficiency values from ***Eq. E12***. On the contrary, a homogeneously mixed state would yield a vanishingly small σ, and thus a high η.

References

1 Jaccard, J., Wan, C. K. & Turrisi, R. The Detection and Interpretation of Interaction Effects between Continuous-Variables in Multiple-Regression. *Multivariate Behavioral Research* **25**, 467-478, doi:10.1207/s15327906mbr2504_4 (1990).

2 Figliola, R. S. & Beasley, D. *Theory and design for mechanical measurements*. (John Wiley & Sons, 2011).

3 Dawson, J. F. & Richter, A. W. Probing three-way interactions in moderated multiple regression: Development and application of a slope difference test. *Journal of Applied Psychology* **91**, 917-926, doi:10.1037/0021-9010.91.4.917 (2006).

4 Aiken, L. S., West, S. G. & Reno, R. R. *Multiple regression: Testing and interpreting interactions*. (Sage, 1991).
